# A Novel Lipopeptaibol Emericellipsin A with Antimicrobial and Antitumor Activity Produced by the Extremophilic Fungus *Emericellopsis alkalina*

**DOI:** 10.3390/molecules23112785

**Published:** 2018-10-27

**Authors:** Eugene A. Rogozhin, Vera S. Sadykova, Anna A. Baranova, Alexey S. Vasilchenko, Vladislav A. Lushpa, Konstantin S. Mineev, Marina L. Georgieva, Alexander B. Kul’ko, Mikhail E. Krasheninnikov, Alexey V. Lyundup, Anastasia V. Vasilchenko, Yaroslav A. Andreev

**Affiliations:** 1 Shemyakin and Ovchinnikov Institute of Bioorganic Chemistry, RAS, ul. Miklukho-Maklaya, 16/10, Moscow 117997, Russia; lushpa@phystech.edu (V.A.L.); konstantin.mineev@gmail.com (K.S.M.); 2 Gause Institute of New Antibiotics, ul. Bolshaya Pirogovskaya, 11, Moscow 119021, Russia; sadykova_09@mail.ru (V.S.S.); anjabaranowa@list.ru (A.A.B.); i-marina@yandex.ru (M.L.G.); 3 Tyumen State University, 6 Volodarskogo str, Tyumen 625003, Russia; avasilchenko@gmail.com (A.S.V.); vasilchenko.av.83@gmail.com (A.V.V.); 4 Moscow Institute of Physics and Technology, Institutskiy per., 9, Dolgoprudnyi 141701, Russia; 5 Lomonosov Moscow State University, 1-12 Leninskie Gory, Moscow 119991, Russia; 6 Moscow Government Health Department Scientific and Clinical Antituberculosis Center, ul. Stromynka, 10, Moscow 107014, Russia; kulko-fungi@yandex.ru; 7 Institute of Molecular Medicine, Advanced Cell Technologies Department, Institute for Regenerative Medicine, Sechenov First Moscow State Medical University, Trubetskaya St. 8, Bldg. 2, Moscow 119991, Russia; krashen@rambler.ru (M.E.K.); lyundup@gmail.com (A.V.L.)

**Keywords:** Peptaibol, emericellipsin A, *Emericellopsis alkalina*, 2D structure, antifungal activity, antibacterial activity, cytotoxic properties

## Abstract

Soil fungi are known to contain a rich variety of defense metabolites that allow them to compete with other organisms (fungi, bacteria, nematodes, and insects) and help them occupy more preferential areas at the expense of effective antagonism. These compounds possess antibiotic activity towards a wide range of other microbes, particularly fungi that belong to different taxonomical units. These compounds include peptaibols, which are non-ribosomal synthesized polypeptides containing non-standard amino acid residues (alpha-aminoisobutyric acid mandatory) and some posttranslational modifications. We isolated a novel antibiotic peptide from the culture medium of *Emericellopsis alkalina*, an alkalophilic strain. This peptide, called emericellipsin A, exhibited a strong antifungal effect against the yeast *Candida albicans*, the mold fungus *Aspergillus niger*, and human pathogen clinical isolates. It also exhibited antimicrobial activity against some Gram-positive and Gram-negative bacteria. Additionally, emericellipsin A showed a significant cytotoxic effect and was highly active against Hep G2 and HeLa tumor cell lines. We used NMR spectroscopy to reveal that this peptaibol is nine amino acid residues long and contains non-standard amino acids. The mode of molecular action of emericellipsin A is most likely associated with its effects on the membranes of cells. Emericellipsin A is rather short peptaibol and could be useful for the development of antifungal, antibacterial, or anti-tumor remedies.

## 1. Introduction

Filamentous fungi are historically known as an excellent source of antimicrobial peptides synthesized by nonribosomal peptide synthetases (NRPSs). However, only a few investigations of alkalophilic fungi have been conducted. Haloalkaliphilic fungi are a unique group of extremophiles that grow optimally in conditions of extreme salinity and alkalinity. High salinity and low nutrient availability lead to unique adaptations within these fungi and may lead to the potential for the discovery of new bioactive molecules. Species in the genus *Emericellopsis* produce a spectrum of peptide antibiotics with antibacterial and antifungal activity. Peptaibols isolated from Emericellopsis species include zervamicins (produced by *E. microspora*) [1,2], bergofungins A and B (produced by *E. donezkii*), bergofungins C and D (produced by *E. salmosynnemata*) [3,4,5], and heptaibin and emerimicines (produced by *E. minima*) [6]. Screening of metabolites of several alkalophilic strains isolated from saline soils has revealed the fungus *Emericellopsis alkalina* strain VKPM F-1428, which demonstrates promising antifungal activity against different fungal taxons [7]. Bioassay-guided fractionation makes it possible to isolate the novel peptaibol, termed emericellipsin A. The details of the purification process, structure elucidation, and antimicrobial and cytotoxic activities of emericellipsin A are reported herein.

## 2. Results and Discussion

**Figure 1:**
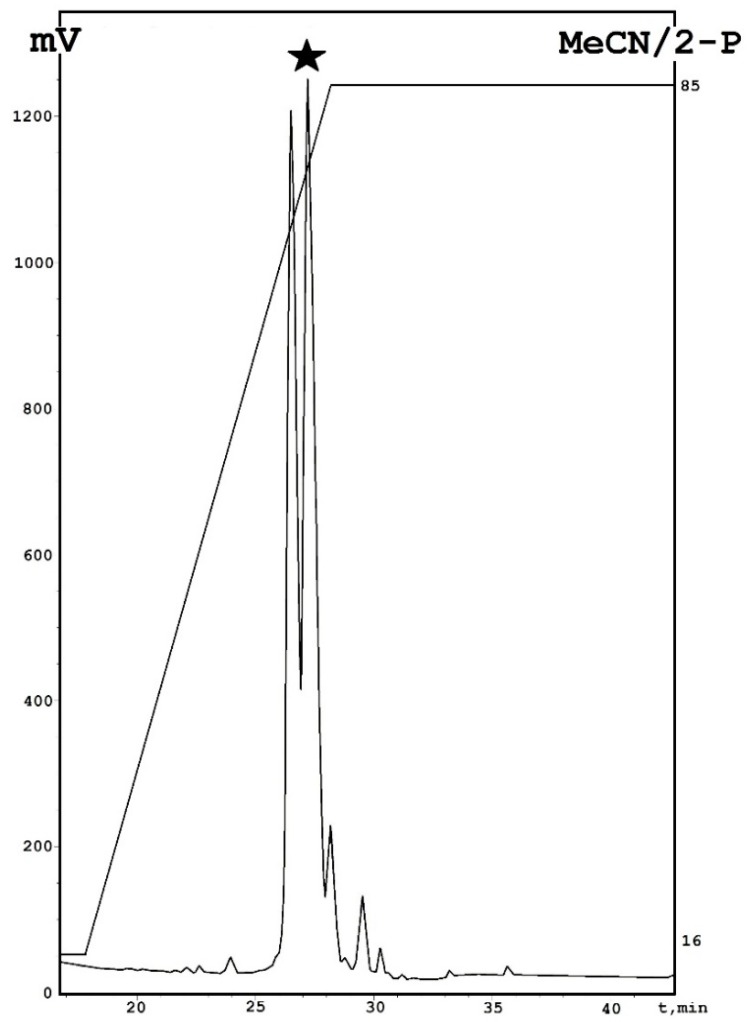
Purification of emericellipsin A by phenyl-modified reversed-phase HPLC. The target peak was marked by a black star. Specific descriptions: MeCN—acetonitrile; 2-P—isopropanol.

Emericellipsin A was isolated from fungal culture liquid as described previously with modification [7]. The scheme includes a combination of ethyl acetate extraction followed by evaporation, dissolving in ethanol and analytical reversed-phase HPLC on a C_18_ phase [7]. One additional purification step, based on analytical phenyl RP-HPLC, was used to obtain the individual component. As result, two different components were found in the previously described active fraction (Figure 1). An antimicrobial assay of these compounds revealed activity for the second peak, which is referred to as emericellipsin A. Mass spectrometry made it possible to identify a monoisotopic molecular mass of 1049.76 Da. The structure of this peptide was determined using NMR spectroscopy.

**Figure 2:**
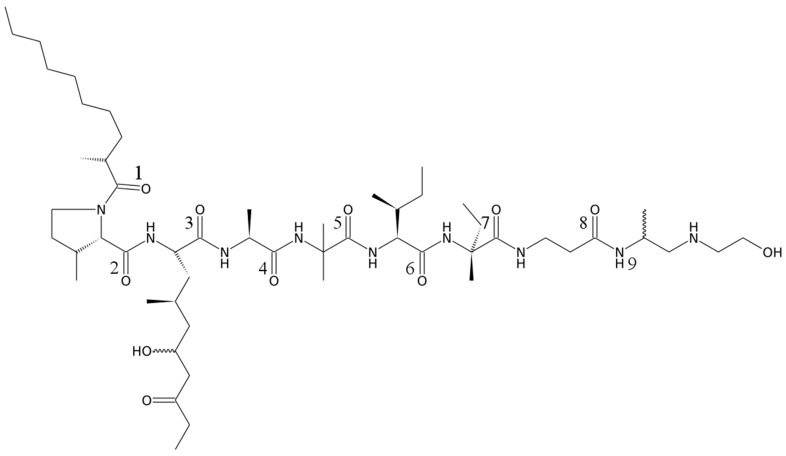
Structure of emericellipsin A determined by NMR spectroscopy. Numbering of amino and fatty acid residues is shown corresponding to the Table S1.

According to NMR spectra, emericellipsin A is a linear polypeptide flanked by the 2-methyldecanoic acid (2MDA) at the *N*-terminus and by *N*-(2-Hydroxyethyl)-1,2-propanediamine at the C-terminus. Peptide contains eight carboxyl and one ketone groups, the presence of eight amino groups and one ternary nitrogen was confirmed based on the 1H-15N HSQC and 1H-15N HMBC spectra. Out of seven amino acid residues, two were conventional (alanine and isoleucine), and other were 3-methylproline (3MP), 2-Amino-4-methyl-6-hydroxy-8-oxo decanoic acid (AHMOD) [8], 2-aminoisobutyrate (AIB), isovaline, and β-alanine (Table S1). NMR spectra revealed the molecular formula C54H99N9O11 with the isotopic molecular mass 1049.746 that agreed with the mass spectra (1049.7568). We used the ROESY spectrum to determine the configuration of stereo centers. In the spectrum, the network of characteristic (i,i+3) and (i,i+4) contacts is observed, which, together intense HN-HN(i,i+1) cross-peaks, suggests that the peptide adopts an α-helix conformation. Taking into account that alanine is in the l-configuration in homologous lipoaminopeptides, such as culicinins A-D [9], the configuration of other amino acids can be easily determined following the NOE contacts. The specified analysis revealed that all amino acids, including the AHMOD, isovaline, and substituted proline are in the l-configuration. Configuration of 2MDA was determined taking into account that, according to the strongest contact between the C2H proton and CδH2 group of 3-methyl-proline, the C1–C2 bond in 2MDA is in the 180° conformation. This allows the straightforward analysis of the network of the ROESY cross-peaks between the C3H2, C2’H3 groups of 2MDA and ProS and ProR protons of CδH2 group of 3-methyl-proline, which reveals the S configuration of C2 stereocenter. Analogously, we managed to determine the S configuration of Cβ in 3MP, which follows from the ROESY cross-peaks between the methyl group/CβH proton and CαH proton of the residue. A more complicated analysis was necessary to determine the configuration of Cγ (C4) stereocenter of AHMOD. Analysis of short distances and J-couplings revealed that χ1 of the residue is in −60° conformation, while χ2 is in 180°. This allowed the stereospecific assignment of CβH2 protons of AHMOD, and ROESY contacts between the Cδ2 methyl group and CβH2 protons reveal the S-configuration of the stereo center. The 4-S configuration of AHMOD was previously confirmed for the other peptaibol [10]. The further analysis of AHMOD configuration is impossible, because the χ3 and following side chain angles are not fixed in the certain conformation and Cδ1H2 protons are not resolved, which leaves the configuration of stereo center 6 (Cε1) undefined. However, 6-R configuration may be expected based on the structures of other AHMOD-containing peptaibols [10]. Similarly, we failed to determine the configuration of C1 center in the C-terminal *N*-(2-Hydroxyethyl)-1,2-propanediamine. The obtained structure is shown in Figure 2 and chemical shift assignments are provided in the Supplementary Materials in the Table S1. Emericellipsin A is a typical representative of so-called lipoaminopeptides or aminolipopeptides subfamily of peptaibols [11]. These peptides are characterized by the presence of alpha-methyl branched fatty acid at the *N*-terminus, followed by the proline derivative at position 2 and AHMOD at position 3.

Most peptaibols isolated from mycelial fungi are represented peptides 10–20 residues long with molecular masses of 1.5–2.0 kDa [12,13]. Emericellipsin A is nine residues long and therefore is more suitable for drug development substance than most previously described peptaibols. Ethyl acetate was used for extraction the active compound from the culture medium after *E. alkalina* A118 strain fermentation. A large fraction of it exhibited significant antimicrobial activity. The most antifungal activity was concentrated in two main peaks only (FIII and FIV) that were consequently purified by analytical RP-HPLC to isolate many components, which were inactive with the exception of emericellipsin A. Interestingly, the total organic extract from the A118 culture medium displayed weak bactericidal activity against the opportunistic Gram-positive bacterium *Bacillus subtilis* [10]. In this study, the antimicrobial activity of emericellipsin A was estimated with MIC values using various collection strains as well as clinical fungal isolates. Co-incubation of microorganisms with emericellipsin A revealed the ability of emericellipsin A to kill only fungi and Gram-positive bacteria; Gram-negative bacteria demonstrated resistance to this compound (Table 1). The bactericidal effect of emericellipsin A is comparable to antifungal action (4.0–32.5 µg/mL) (Table 2); all evaluated Gram-negative strains were insensitive at concentrations below 300 µg/mL.

It is interesting that the same dependence in antimicrobial activity was demonstrated by the reference positive control, vancomycin, which belongs to the group of glycopeptide antibiotics [14] and is structurally dissimilar to emericellipsin A. The same effect was demonstrated for peptaibol emerimicin IV, which was isolated from *Emericellopsis minima* and displays bactericidal activity towards methicillin-resistant *S. aureus* and vancomycin-resistant *Enterococcus faecalis* (Gram-positive species); Gram-negative *E. coli* was resistant [15]. In general, the primary mechanism of the peptaibol action is associated with the disruption of cellular membranes [1,16].

Larger peptaibols with more than 15 amino acids can form stable helical structures in the membrane [17]. These helices can associate in oligomers and form ion channels in the membrane. Shorter peptaibols are less membrane active and therefore the mode of their action is more complex. Their action may be a combination of membrane-disrupting activity and an effect on different molecular targets [9,18]. Nevertheless, short peptaibols could affect the membrane via a variety of mechanisms: they could form end-to-end bundles within the bilayer, thereby effectively doubling their length perpendicular to the bilayer, or they could form membrane-associated aggregates or act via a detergent-like mechanism. Therefore, the properties of peptaibols allow them to exhibit differential activities when targeting different membrane lipid types. They accordingly affect organisms with different membrane characteristics than their own [19,20].

We evaluated the ability of emericellipsin A to disrupt bacterial barrier structures. Using DNA-binding stains SYTO9 and propidium iodide (PI), we investigated the dynamics of their intracellular accumulation in real time. This stained mixture is actively used for investigation of AMP’s mode of action. Often, this approach allows obtaining the unique information about the peculiarities of the action of peptides, which is not available to other methods, for example, bacteriological [21,22]. The green fluorescent SYTO 9 is a relatively small molecule (~400 Da) which is able to influx trough non-damaged bacterial membranes, while PI is a large molecule (668 Da) that penetrates only into damaged cellular barrier structures [23]. The emission properties of the stain mixture bound to DNA change due to the displacement of one stain by the other and quenching by fluorescence resonance energy transfer [24].

Earlier we successfully performed this approach for investigation of mode of action of the various antimicrobial peptides [25]. We showed that this effect is really occurred when PI was able to influx into the cells via a disordered barrier following displacement of the SYTO9 from the DNA [26].

**Figure 3:**
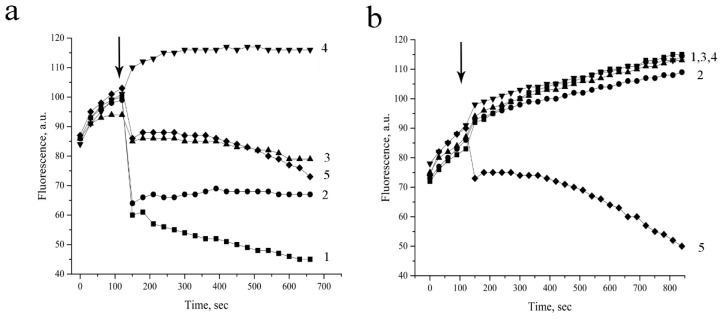
Dynamic of permeation of SYTO 9 into *S. aureus* 209 P (**a**) and *E. coli* MG 1655 (**b**) cells treated with emericellipsin A. Designations: 1—75 μg/mL; 2—32.3 μg/mL; 3—16 μg/mL; 4—negative control; 5—positive control. If bacterial membranes are permeabilized, PI penetrates into the cell. What follows is SYTO 9 getting displaced from DNA, which leads to a decrease in luminescence intensity in a green region of the spectrum. Pure-water and 20% alcohol served as negative and positive controls, respectively. Arrows show the time of test-substance.

The addition of the peptaibol to *S. aureus* cells led to the immediate quenching of SYTO9 fluorescence (Figure 3).

This event suggests disruption of the *S. aureus* cytoplasmic membrane under the treatment. In turn, mixing of emericellipsin A with *E. coli* cells did not change the kinetics of the SYTO9 fluorescence, suggesting that only low-molecular weight compounds are able to transfer into the cell. However, emericellipsin A can affect the cell walls of Gram-negative bacteria. A breach in the outer membrane of Gram-negative bacteria was detected using a hydrophobic fluorescent probe. 1-*N*-phenylnaphthylamine (NPN) is a hydrophobic, neutrally charged substance normally impermeable into the outer membrane, but if the molecules of NPN internalize in phospholipid environments, its fluorescence strongly increases [27,28].

The addition of various concentrations of emericellipsin A to *E. coli* MG 1655 led to an increase in the fluorescent intensity of the NPN in a dose-dependent manner (Table 2). The maximum response was observed at a concentration of 30 µg/mL.

Therefore, the mode of action of emericellipsin A is associated with the disruption of the bacterial cytoplasmic membrane, which took place within several minutes and led to the death of the Gram-positive bacteria. At the same time, the outer membrane of Gram-negative bacteria also takes a hit by protecting the cytoplasmic membrane from peptaibol molecules. The peptide studied ensures the survival of *E. coli* but could affect their virulence in, for example, biofilm formation.

Emericellipsin A exhibited broad-spectrum antifungal activity in the agar diffusion assay; it inhibited growth of all Candida species and the filamentous fungi *A. niger* ATCC 16404 and *A. fumigatus* KBP F24 at a concentration of 40 μg/per disc. Different levels of susceptibility were demonstrated for the clinical multi-resistant isolates of *Aspergillus* that indicated strain-specific sensitivity to the peptaibol. More precisely, the peptaibol was effective against *A. niger* 219, *A. fumigatus* 163, *A. flavus* 905 and was slightly effective against *A. tereus* 1133. As shown in Table 3, a moderate inhibition effect of the peptaibol was observed against all isolates of the *Aspergillus* genus (MIC values of 4 μM), and strong antifungal activity was observed against drug-resistance isolates of *C. tropicales* 1402 and *C. albicans* 1582 with the same MIC value of 2 μM. It is noteworthy that the clinical yeast isolates were more susceptible to the peptaibol than the *Aspergillus* isolates. This finding agrees well with the existing data on fungal peptaibols’ spectrum of activity [29,30,31]. It is noteworthy that the clinical yeast isolates were more susceptible to the peptaibol than the *Aspergillus* isolates.

**Figure 4:**
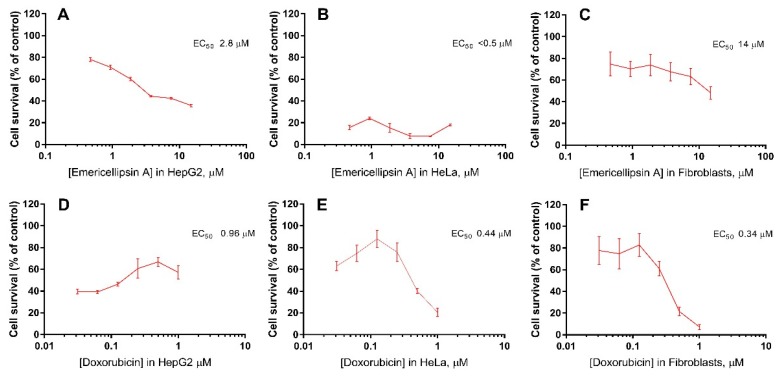
Comparative cytotoxic activity of emericellipsin A (**A**–**C**) and doxorubicin (positive control) (**D**–**F**): HepG2 tumor cell line (**A**,**D**); HeLa tumor cell line (**B**,**E**) and human fibroblasts (**C**,**F**).

It is well known that many peptaibols are cytotoxic and that some of them can suppress tumor cell lines much better than normal cells by inducing calcium-mediated apoptosis [32]. In vitro assays of emericellipsin A exhibited selective cytotoxic activity against HepG2 and Hela cell lines (EC_50_ 2.8 and < 0.5 μM, respectively) (Figure 4).

This result is consistent with the standard antitumor antibiotic doxorubicin, which has an EC_50_ value of 440 nM. In a fibroblast toxicity test, emericellipsin A exhibited less cytotoxic activity than doxorubicin (EC_50_ 14 and 0.34 μM, respectively). Therefore, it is less toxic to normal cells than doxirubicin (~40 times), but it yields a more potent cytotoxic effect on tumor cell lines. Emericellipsin A can be considered to be an effective anti-tumor substance. Peptaibol culicinin D isolated from the entomopathogenic fungus *Culicinomys clavisporus* strain LL-12I252 was previously described as potent anticancer compound [9]. This molecule has been tested in vitro MTT assays to inhibit MDA468 (PTEN −/−) and MDA435 (PTEN +/+) breast tumor lines at a range of active concentrations ranging from 1 ng to 10 µg/mL. Interestingly, there was no linear dose-dependent response, and EC_50_ was determined at a wide range of concentrations that are differed over two–three orders of magnitude in terms of the tumor line tested [9]. Emericellipsin A produced a standard concentration dependence of activity on HepG2 cells and significantly decreased the survival of HeLa cells at all tested concentrations.

## 3. Materials and Methods

### 3.1. Fungal Strain and Cultivation

The strain A118 of *Emericellopsis alkalina* Bilanenko and Georgieva was isolated from alkaline soil on the edge of the Zheltyr Lake, Kulunda steppe, Russia. It was deposited at the Collection of Fungi from Extremophilic Habitat Department of Mycology and Algology Biological Faculty Lomonosov Moscow State University and All-Russian Collection of Industrial Microorganisms (Moscow, VKPM F-1428). Species identification was conducted by molecular-genetics methods based on sequence data of ITS rDNA, LSU rDNA, SSU rDNA, TEF-1α, β-tub, and RPB2 in Laboratory of Genetics, Plant Sciences Group, Wageningen University, the Netherlands. The DNA sequences were deposited to GenBank: ITS1, ITS2, 5.8S (ID: KC987155.1); LSU rDNA (ID: KC987230.1); SSU rDNA (ID: KC987193.1); TEF-1α (ID: KC998977.1); β-tub (ID: KC987117.1); and RPB2 (ID: KC999014.1) [33]. Large-scale cultures, used for isolation of peptaibols, were grown in 20 Erlenmeyer flasks (size 500 mL) resulting in a total volume of 2.0 L on special alkaline medium (pH 10.5) that consisted of (per liter of tap water): salts: Na_2_CO_3_–24 g, NaHCO_3_–6 g, NaCl–6 g, KNO_3_–1 g, K_2_HPO_4_–1 g; malt extract–200 mL, yeast extract–1 g. Each culture flask was inoculated with a 10 mm agar plug of colonized fungus and incubated for 14 days at 25 °C at stationary condition without agitation.

### 3.2. Microorganisms

The spectrum of antifungal activity was evaluated against fungi from the Collection of Cultures for the Search for New Antibiotics (Gauze Scientific Research Institute, Russia). We used mold fungi belonging to *Aspergillus* genus, i.e., *A. fumigatus* KBP F24 and *A. niger* INA 00760 and *Candida* — *C. albicans* ATCC 2091 and *C. tropicalis* INA 00763. Pathogenic multi-drug resistance fungi were taken from the Collection of Moscow Municipal Scientific Practical Center of Tuberculosis Control. *A. tereus* 1133 m, *A. flavus* 905 m, *A. ochraceus* 497, *A. fumigatus* 163, *A. niger* 219, *C. albicans* 1582, *C. glabrata* 1402 m, *C. tropicalis* 1402, and *C. krusei* 1308 were isolated from patients having invasive pulmonary aspergillosis and oropharyngeal HIV-positive patients. All clinical fungal cultures have demonstrated in vitro resistance to commercial azoles. Used bacterial strains were obtained from commercially available culture collections.

### 3.3. Isolation and Purification of Emericellipsin A

Isolation of the target peptaibol from the culture liquid was carried accordingly described earlier [7]. Rechromatography of the emericellipsin A-containing fraction was performed on a Synergi Polar-RP (250 × 4.6 mm 4 µm 80 Å) analytical column (Phenomenex, Torrance, CA, USA) in a linear gradient of acetonitrile/isopropanol (4:1, *w*/*w*) mixture with 0.1% trifluoriacetic acid (TFA) from 16 to 85% for 45 min, flow rate of 1 mL/min and detection of absorbance at 210 nm.

### 3.4. Mass Spectrometry

The peptide sample was analyzed with an LC-MS/MS system (Agilent Technologies, Santa Clara, CA, USA) consisting of a nanopump (G2226A, Agilent) with a four-channel micro vacuum degasser (G1379B, Agilent), a microfluidic chip cube (G4240-64000, Agilent) interfaced to a Q-TOF mass spectrometer (6530, Agilent), a capillary pump (G1376A, Agilent) with degasser (G1379B, Agilent), and an auto-sampler with thermostat (G1377A, Agilent). All modules were controlled by Mass Hunter software (version B.06.00, Agilent). A microfluidic reversed-phase HPLC chip (Zorbax 300SB-C_18_, 5-μm particle size, 0.75 × 150 mm) was used for peptide separation. A mixture of 96.9% water, 3% acetonitrile, and 0.1% formic acid (*v*/*v*) was used as the sample loading solution and solvent. Buffer B was 99.9% ACN, 0.1% formic acid (*v*/*v*). Samples were loaded on a trap-column at a flow rate of 3 µL/min for 5 min and eluted through a separation column at a flow rate of 300 nL/min. The gradient was from 15 to 85% of buffer B within 30 min.

### 3.5. NMR Spectrometry

All NMR spectra were recorded on the Avance Bruker 800 spectrometer (Bruker Biospin, Rheinstetten, Germany). The concentration of compound was approximately three mg/mL. To determine the structure of emirecellipsin A, we employed the conventional NMR-based approach, involving the analysis of 2D COSY, 2D 1H-13C HSQC, 2D 1H-15N HSQC, 2D 1H-13C HMBC, 2D 1H-15N HMBC, and 2D 1H-13C HSQC-TOCSY spectra. 2D ROESY (200 ms mixing time) was recorded to determine the configuration of stereo centers.

### 3.6. Antibacterial Activity

Determination of the minimum inhibitory concentration (MIC) was carried out by conventional broth microdilution methods based on the Clinical and Laboratory Standards Institute (CLSI) adapted for antimicrobial peptides [34]. The overnight cultures of the test strains were diluted in Muller Hinton broth (HiMedia, Mumbai, India) in order to obtain 10^6^ CFU/mL. Prepared inoculums were mixed with two-fold dilutions of emericellipsin A and incubated for 24 h in 96-wells microtiter plate (Eppendorf, Hamburg, Germany). The indolicidin (Research Institute of Highly Pure Biopreparations, Saint Petersburg, Russian Federation), vancomycin (Sigma-Aldrich, St. Louis, MO, USA), and norfloxacin (Sigma-Aldrich) were used as positive controls. After incubation, the optical density of planktonic cells was assessed by reading the absorbance data at 620 nm. These data were obtained by the IEMS MF spectrophotometer (Labsystems, Vantaa, Finland). Antimicrobial activity of emericellipsin A was indicated by the minimal inhibitory concentration, which was defined as the lowest dose at which no visible growth was detected. Determination of bactericidal activity of emericellipsin A was performed by plating of the treated bacteria from the wells on agar medium (Muller Hinton, HiMedia). Following incubation, CFU counting was conducted.

### 3.7. Permeabilization of the Bacterial Cell Wall. Evaluation of the Outer Membrane Disturbance

The bacterial cells of *E. coli* MG 1655 were precipitated by centrifugation at 7000 g for 10 min and re-suspended in five mmol/mL HEPES (pH 7.5) buffer to an optical density (OD_620_) of 0.1. Bacterial suspension was mixed with emericellipsin A taken at seven, 15, and 30 μg/mL. After incubation for one hour, the reaction mixture was subjected to a quartz quiet contained 10 μmol/L of the 1-*N*-phenylnaphthylamine (NPN) (Sigma-Aldrich). The control solvents contained the following: (a) Buffer and 10 μmol/L of NPN; (b) 10 μmol/L of NPN and cells without emericellipsin A; and (c) 10 μmol/L of NPN and cells treated with 0.5 mol/L EDTA (positive control). After incubation with NPN for 5 min, the spectra of fluorescence were recorded using a spectrometer Fluorat-02 Panorama (Lumex, St. Petersburg, Russia) at an excitation of 350 nm and an emission of 380–500 nm. The results are expressed as NPN uptake factors. The NPN uptake factor was calculated as a ratio of background-corrected (subtracted by the value in the absence of NPN) fluorescence values (at the point of fluorescence maximum) of the bacterial suspension (the cells which were treated and non-treated cells) and of the buffer, respectively.

### 3.8. Permeabilization of the Bacterial Cytoplasmatic Membrane

The LIVE/DEAD BacLight Bacterial Viability Kit (Molecular Probes, Eugene, OR, USA) was used to evaluate the cytoplasmatic membrane integrity of *S. aureus* 209 P and *E. coli* MG 1655 according to the manufacture’s protocol. Measurement of SYTO 9 fluorescence kinetic was performed using the Infinite F200 pro plate reader (Tecan, Salzburg, Austria), at 485 nm emission and 535 nm of excitation wavelength. 20% ethanol and pure water were used as positive and negative controls, respectively.

### 3.9. Antifungal Activity

Preliminarily spectrum of antifungal activity of the compound was evaluated in vitro by disc diffusion assay. Yeasts and fungal cells (100 μL; approximately 10^6^ CFU/mL) were spread on potato-dextrose agar (PDA) (Sigma, Ronkonkoma, NY, USA) plates. Whatman filter paper No. 1 discs (6 mm in diameter) impregnated with the concentration at 40 μg/disc were placed on the plates, and then the plates were incubated at 37 °C for 24 h. The total diameter of the inhibition zone was measured by hand with a ruler. Minimum inhibitory concentrations were detected in a serial dilution assay with the purified compounds following the previously described protocol. The tests were carried out by taking a 100 μL stock solution of each component in a two-fold serial dilution at concentrations in the range from 0.5 to 16 μg/mL in DMSO (Merck, Kenilworth, NJ, USA). The assays were conducted in 96-well microtiter plates (BioCell Technology, Newport Beach, CA, USA) in RPMI 1640 (PanEco, Moscow, Russia) medium without the addition of Na_2_CO_3_. In the case of positive control, amphotericin B was used. The solvent medium was used as a negative control. MIC values were defined as the lowest concentration of compounds at which the microorganisms tested did not demonstrate visible growth after 48 h of incubation. Each experiment was carried out in triplicate.

### 3.10. Cytotoxic Assays

The cytotoxic activity was investigated using the MTT-test method. The cytotoxicity of the emericellipsin A was evaluated in two human cell tumor lines: HepG2 (human liver cancer cell line) and Hela (cervical cancer cell line). Human postnatal fibroblasts were used as a normal cell line, and doxorubicin only was used as a positive control. All cells were cultured as adherent monolayers in flasks supplemented with 10% fetal bovine serum, L-glutamine (2 mM), penicillin (100 unit/mL), and streptomycin (100 μg/mL), in a humidified 37 °C incubator supplied with 5% CO_2_. Briefly, cells were harvested with trypsin and dispensed into 96-well microtiter assay plates at ~20 × 10^3^/sm^2^ (30% from a monolayer), after which they were incubated for 12 h at 37 °C with 5% CO_2_ (to allow cells to attach as adherent monolayers). Test compound was dissolved in 20% DMSO in PBS (*v*/*v*), and aliquots (10 μL) applied to cells over a series of final concentrations ranging from 0.1 to 1 μM. After 72 h of incubation, 3-(4,5-dimethylthiazol-2-yl)-2,5-diphenyltetrazolium bromide (MTT) saline solution (1 mg/mL, 50 μL) was added to each well and microtiter plates were incubated for a further four h at 37 °C with 5% CO_2_. After final incubation the supernatant was discarded, DMSO (150 μL) was added, and the absorbance at 570 nm was measured with a Bio-Rad 680 microplate reader (Bio-Rad Laboratories, Hercules, CA, USA). All experiments were repeated three times and each time in triplicate.

## 4. Conclusions

In conclusion, emericellipsin A is a novel peptaibol with high antifungal (fungicidal) and antibacterial activity and attractive antitumor potential. It is shorter than other peptaibols with similar biological activities and therefore more suitable for drug development. In particular, despite the absence of a bactericidal effect detected towards Gram-negative bacteria, the peptaibol displayed anti-biofilm formation activity. The substantial antifungal activities of peptaibol indicated for clinical Candida and Aspergillus isolates highlight its potential for use as a novel antifungal agent active against drug-resistant fungi. Additional investigations of emericillipsin A agent would be of great interest. These properties could be quite valuable to decrease the virulence potential of opportunistic and pathogenic microbiota with non-selective action on fungal and bacterial populations.

## Figures and Tables

**Table 1:** The antibacterial activity of emericellipsin A against bacteria.

Microorganisms	Strains	MIC, μg/mL			
		Indolicidin	Emericellipsin A	Vancomycin	Norfloxacin
Gram-negative	*Escherichia coli* MG1655	25	>300	>200	0.08
	*Salmonella enterica* ATCC 14028	100	>300	>200	1.25
	*Pseudomonas aeruginosa* ATCC 27853	100	>300	>200	2.5
Gram-positive	*Bacillus cereus* ATCC 14893	12.5	16	12.5	>28
	*Staphylococcus aureus* FDA 209 P	12.5	4	3.1	0.31
	*Listeria monocytogenes* EGDe	3.25	32.5	0.38	1.75

**Table 2:** 1-*N*-phenylnaphthylamine (NPN) uptake of *Escherichia coli* MG 1655 induced by permeabilizers.

Samples	NPN Uptake Factor ± SD
*Escherichia coli* MG1655	1.5 ± 0.05
*Escherichia coli* MG1655 treated with 0.5 M EDTA	1.83 ± 0.1
*Escherichia coli* MG1655 treated with 7 µg/mL of emericellipsin A	2.0 ± 0.1
*Escherichia coli* MG1655 treated with 15 µg/mL of emericellipsin A	2.3 ± 0.2
*Escherichia coli* MG1655 treated with 30 µg/mL of emericellipsin A	4.7 ± 0.2

**Table 3:** Minimum Inhibitory Concentrations (MIC) of the emericellipsin A against fungi, μg/mL.

Microorganism	Emericellipsin A	Fluconazol	Amphotericin B
*C. tropicales* 1402	2	R *	1.0
*C. albicans* 1582	2	R	1.0
*C. albicans* ATCC14053	2	0.25	0.25
*A. niger* ATCC 16404	4	1.0	1.0
*A. niger* 219	4	R	0.5
*A. fumigatus* 163	4	R	1.0
*A. flavus 905*	4	R	0.5

* R—resistant.

## Supplementary Materials

The following are available online at http://www.mdpi.com/1420-3049/23/11/2785/s1. Table S1: Chemical shifts and HMBC correlations observed in the NMR spectra of emericellipsin A.
