# Supplementary material for: A Novel Lipopeptaibol Emericellipsin A with Antimicrobial and Antitumor Activity Produced by the Extremophilic Fungus Emericellopsis alkalina
Source: Molecules. 2018 Oct 27;23(11):2785. doi: 10.3390/molecules23112785 (PMC6278523; doi:10.3390/molecules23112785)
Supplement: Supplementary file 1 [file molecules-23-02785-s001.pdf]

Table S1. Chemical shifts and HMBC correlations observed in the NMR spectra of emericellipsin A.

№	Amino acid	Position	13C/15N	1H	HMBC*
		C1	176.05	-	-
		C2	37.159	2.627	C1,C3,C3′,C4
		C3	33.833	1.27/1.51	C1, C2, C3, C3′, 5
		C3′	17.435	1.022	C1, C2, C3
		C4	27.084	1.196/1.276	C5
1	(2R)-2-methyl decanoic acid	C5	29.542	1.237	C4, C6, C7
		C6	29.382	1.238	C4, C5, C7, C8
		C7	29.07	1.239	C5, C6, C8
		C8	31.729	1.237	C7, C9, C10
		C9	22.556	1.262	C7, C8, C10
		C10	14.421	0.856	C8, C9
		C	172.9	-	-
		Cα	67.9	3.72	1-C1, C, Cβ, Cγ1, Cγ2, Cδ1
		Cβ	37.7	2.2	Cα, Cγ1, Cγ2, Cδ1
2	(3R)-3-methylproline	Cγ1	32.7	1.577/2.08	N, Cα, Cβ, Cγ2, Cδ1
		Cγ2	17.9	1.056	Cα, Cβ, Cγ1
		Cδ1	46.7	3.62/3.685	Cβ, Cγ1
		N	132.000	-	-
		C	174.067	-	-
		Cα	52.617	4.126	2-C, C, N, Cβ, Cγ
		N	115.241	8.069	2-C, C, Cα, Cβ
		Cβ	37.586	1.449/1.731	N, C, Cα, Cγ, Cδ1, Cδ2
		Cγ	26.587	1.663	Cα, Cβ, Cδ1, Cδ2, Cε1
		Cδ1	45.186	1.294	Cγ, Cβ, Cδ2, Cε1, Cξ1
3	(2S,4S)-2-Amino-4-methyl-6-hydroxy-8-oxodecanoic acid (AHMOD)	Cδ2	19.88	0.862	Cβ, Cγ, Cδ1
		Cε1	65.402	3.988	Cγ, Cδ1, Cξ1, Cη1
		Cξ1	50.634	2.445	Cδ1, Cε1, Cη1, Cθ1
		Oξ2	-	4.623	Cγ, Cδ1, Cε1, Cξ1
		Cη1	210.532	-	-
		Cθ1	36.425	2.445	Cη1, Cι1
		Cι1	7.917	0.907	Cη1, Cθ1
4	L-Alanine	C	173.798	-	-

		Cα	51.023	3.993	C, N, Cβ
		N	120.328	7.85	C, Cα, Cβ
		Cβ	16.964	1.313	C, N, Cβ
		C	173.791	-	-
		Cα	56.317	-	-
5	2-Aminoisobutyric acid	N	126.292	7.873	4-C, C, Cα, Cβ1, Cβ2
		Cβ1	25.920	1.344	C, N, Cα, Cβ2
		Cβ2	24.414	1.369	C, N, Cα, Cβ1
		C	171.167	-	-
		Cα	60.042	3.783	5-C, C, Cβ, Cγ1, Cγ2
		N	110.329	7.271	5-C, C, Cα, Cβ
6	L-Isoleucine	Cβ	35.613	1.865	Cα, Cγ1, Cγ2, Cδ1
		Cγ1	25.419	1.126/1.513	Cα, Cβ, Cγ2, Cδ1
		Cγ2	15.736	0.839	Cα, Cβ, Cγ1
		Cδ1	11.542	0.810	Cβ, Cγ1
		C	174.495	-	-
		Cα	59.901	-	-
		N	125.328	7.298	6-C, C, Cα, Cβ1, Cβ2
7	L-isovaline	Cβ1	30.714	1.773	N, C, Cα, Cβ2, Cγ1
		Cβ2	20.887	1.326	N, C, Cα, Cβ1, Cγ1
		Cγ1	8.283	0.743	Cα, Cβ1
		C	171.995	-	-
		Cα	36.394	3.114/3.483	7-C, C, Cβ
8	β-alanine	Cβ	36.601	2.223/2.335	N, C, Cα, 7-C
		N	107.479	7.523	7-C, Cα, Cβ
		N1	127.477	7.637	8-C, C1, C2’
		C1	42.000	4.150	8-C, C2’,
		C2	49.720	3.030	C1, N3
		C2’	18.57	1.118	C1
9	N-(2-Hydroxyethyl)-1,2-propanediamine	N3	39.02	8.155/8.339	-
		C4	52.499	3.029	-
		C5	56.745	3.675	C4
		O6	-	5.189	C4, C5

*key cross-peaks between the current proton and carbons/nitrogens, observed in HMBC spectra.
